# Modeling and Optimization of LoRa Networks under Multiple Constraints

**DOI:** 10.3390/s23187783

**Published:** 2023-09-10

**Authors:** Hui Zhang, Yuxin Song, Maoheng Yang, Qiming Jia

**Affiliations:** Tianjin Key Laboratory of Optoelectronic Sensor and Sensing Network Technology, Nankai University, Tianjin 300350, China

**Keywords:** LoRa network, data extraction rate, collisions, network energy consumption, integer programming

## Abstract

With the access of massive terminals of the Internet of Things (IoT), the low-power wide-area networks (LPWAN) applications represented by Long Range Radio (LoRa) will grow extensively in the future. The specific Long Range Wide Area Network (LoRaWAN) protocol within the LoRa network considers both low power consumption and long-range communication. It can optimize data transmission to achieve low communication latency, ensuring a responsive system and a favorable user experience. However, due to the limited resources in LoRa networks, if certain terminals have heavy traffic loads, it may result in unfair impacts on other terminals, leading to increased data transmission latency and disrupted operations for other terminals. Therefore, effectively optimizing resource allocation in LoRa networks has become a key issue in enhancing LoRa transmission performance. In this paper, a Mixed Integer Linear Programming (MILP) model is proposed to minimize network energy consumption under the maximization of user fairness as the optimization goal, which considers the constraints in the system to achieve adaptive resource allocation for spreading factor and transmission power. In addition, an efficient algorithm is proposed to solve this optimization problem by combining the Gurobi mathematical solver and heuristic genetic algorithm. The numerical results show that the proposed algorithm can significantly reduce the number of packet collisions, effectively minimize network energy consumption, as well as offering favorable fairness among terminals.

## 1. Introduction

With the rapid development of the Internet of Things (IoT), Massive Machine-Type Communication (MMTC) [1] plays a crucial role in connecting a vast number of devices and enabling intelligent services. The emergence of MMTC is driven by the explosive growth of IoT devices and the increasing demand for low-power and long-distance transmission. According to predictions, the number of MMTC terminals connected via wireless communication will exceed 30 billion by 2025 [2], which demonstrates the immense potential and wide-ranging applications of the MMTC in the future development of the IoT [3]. Compared to traditional Internet connectivity, the MMTC generally focuses on connecting a large number of machines and IoT devices, spanning various industries and domains such as smart cities, intelligent transportation, industrial automation, and agricultural monitoring. However, the conventional IoT connectivity technologies based on cellular systems usually struggle to meet the requirements of the MMTC applications for low power consumption and independent networking, which has led to the rise of low-power wide-area networks (LPWAN) technologies, bridging the technological gap between short-distance communication and cellular technologies [4]. The LPWAN technologies offer characteristics such as low power consumption, long-distance transmission, and wide coverage, providing an ideal communication solution for the MMTC [5]. Through LPWAN technologies, it helps to realize the intelligent interconnection of massive devices, efficient data transmission, and low-power operation of terminals [6].

In particular, among the existing LPWAN, the Long Range Wide Area Network (LoRaWAN) stands out with significant advantages in large-scale IoT applications [7]. The optimization of the LoRaWAN protocol aims to improve data transmission performance and provide a favorable user experience. It can be achieved by adjusting the transmission parameters of LoRa terminals, using adaptive data rate mechanisms, implementing collision detection and conflict management strategies, and defining different QoS requirements for different types of data traffic. The LPWAN radio access technologies aim to provide cost-efficient and energy-efficient communication across massive deployments of autonomous transducers and have lately experienced tremendous growth [8]. It can connect thousands of devices and provide long-range, low-power communication capabilities.

The Long Range Radio (LoRa) is a typical LPWAN technology that works in the frequency band below 1 GHz. It can be independently networked in the license-free frequency band, and provide low power consumption and long-range communication through unique Chirp spread spectrum modulation [9]. The frequency range of LoRa technology is 486.3∼487.7 MHz. LoRa has strong flexibility in radio parameter configuration, which can reduce network energy consumption, improve radio coverage, and reduce radio interference and error rate by adjusting parameters such as spreading factor, etc. [10].

**Figure 1:**
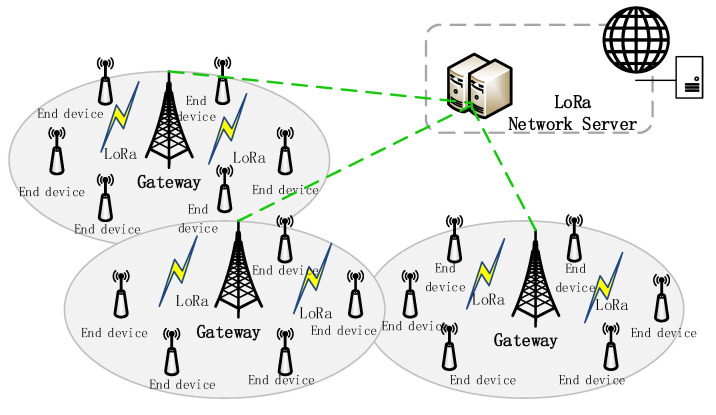
Example of LoRa networks.

LoRaWAN is an open standard promoted by the LoRa Alliance, which defines the medium access control and network management protocol of the LoRa network. LoRaWAN provides three different terminal classes: Type A terminals transmit according to the Aloha protocol, with the lowest power consumption; Type B terminals periodically listen to beacons from the gateway; Type C terminals continuously listen to downlink incoming frames, and consume the highest power consumption [11]. LoRa networks usually adopt a star topology, as shown in Figure 1. The LoRa network architecture is divided into several parts: terminal, gateway, and network server. Generally, data transmission can be carried out between LoRa terminals and gateways through LoRa wireless technology, whereas communication protocols such as TCP/IP or UDP/IP can be used between gateways and network servers to connect through the internet. The network server can manage multiple LoRa gateways and act as a gateway for LoRa terminals to connect to the IoT cloud platform. The radio signal transmitted by the terminal is received by the gateway, which then forwards the data packet to a web server for further processing [10]. In the uplink, the terminal initiates a communication request, and after each uplink transmission, the terminal can receive the data packet and confirmation information sent by the network server.

### 1.1. Related Works

Many existing works generally focused on the performance of LoRa networks, and analyzed from the perspective of communication link, such as scalability [12], performance limitation [13], etc. However, the performance of LoRa terminals depends not only on the communication link, but also on how each gateway manages resources. In the actual LoRa networks, resource allocation is crucial due to a range of issues, including limited network capacity, unfair resource utilization, data conflicts and collisions, energy consumption, and the lifespan of terminals.

Especially, the spreading factor and transmission power are important metrics for adjusting data transmission parameters in the resource allocation of LoRa networks. The spreading factor determines the bandwidth and transmission rate of data, whereas the transmission power determines the transmission distance and reliability of data packets. Although a high spreading factor may reduce bandwidth and data transfer rates, longer communication distances can still be achieved. The spreading factor refers to the expansion of the original signal into a wider frequency band when sending data, and a higher spreading factor means a wider frequency band, so that the interference signal is dispersed, which helps to reduce the interference of data in the transmission process and makes the data packet transmitted over a long distance. At the same time, the high spreading factor allows the network to operate under a relatively low signal-to-noise ratio, and in the process of long-distance transmission, the signal may become very weak due to path loss, but the high spreading factor still allows the receiver to correctly decode and identify these weak signals. The higher transmission power can increase the propagation range of the signal, improve the successful transmission rate of the data packet over a long distance, and improve the reliability of the packet to a certain extent when the signal is weakened. However, excessive transmission power will increase the energy consumption of the terminal and introduce interference, thereby reducing the reliability of the packet. Therefore, the requirements of data transmission rate, distance, and reliability can be effectively balanced by selecting the appropriate spreading factor and transmission power. As a special case, using a higher spreading factor in LoRa networks helps to provide a longer transmission distance but with a lower transmission rate, whereas using a higher transmission power helps to increase the transmission distance and signal penetration but at the cost of higher energy consumption. In addition, the automatic data rate (ADR) technology of LoRa networks helps to optimize the transmission parameters of the terminal devices, including spreading factor and transmission power, based on factors such as data packet quality and network congestion.

On the contrary, the use of unreasonable spreading factors and transmission power in LoRa networks, as well as the lack of ADR technology, may lead to increased unfair resource utilization, data conflicts and collisions, increased energy consumption, and shortened lifespan of terminals. Therefore, in the case of limited resources, it is necessary to select an appropriate spreading factor and transmit power, and utilize the ADR technology to ensure the stability, fairness, and efficiency of LoRa networks, so as to improve the utilization of network capacity.

Moreover, some other existing works have analyzed the effect of LoRa transmission parameters on network performance, such as spreading factor, bandwidth, coding rate, and transmission power, etc., improved LoRa performance through single resource optimization [14]. Moreover, there are adaptive mechanisms considering the configuration of LoRa network communication parameters in dense IoT scenarios, and some parameter assignment algorithms have been proposed. For example, a LoRa terminal can configure its parameters according to the link budget, energy efficiency, network load, etc. [15]. Generally, most of the existing works focused on optimizing a single radio parameter in LoRa to improve network performance. The representative work was as follows.

#### 1.1.1. Spreading Factor (SF)

In recent years, the SF has become a popular parameter for LoRa resource allocation optimization. We take the EXPLoRa-SF algorithm [16] as an example, which determined the percentage of terminals operating for each SF based on the Received Signal Strength Indicator (RSSI), used an ordered water injection method to allocate SFs and balanced the airtime of the data package transmitted by terminals in each SF group. In addition to RSSI, the path loss was also used as a measure to optimize the allocation of SF. The terminals were ordered by the estimated path loss distance to the gateway, and the terminals closest to the gateway were assigned the lowest SF. By optimizing the SF distribution, the average packet error rate of users far from the gateway was reduced [17]. Moreover, the multi-gateway configuration has also become a consideration for allocating SF, and a distributed SF allocation algorithm was proposed based on several criteria facing multi-gateway deployments that effectively allocated SF to progressively joining terminals [18]. Particularly, linear programming has become an important tool for optimizing SF allocation, for example, some works optimized SF allocation by using an integer linear programming model to maximize the number of serving terminals [19]. Moreover, some works adopted an intelligent assignment strategy in SF assignment based on machine learning, which was optimized by using support vector machine and decision tree classifier machine learning. This mechanism usually started from learning the transmission behavior of terminals, and the network server assigned SFs according to its prediction of collisions [20]. In general, the adaptive SF allocation algorithm often had great flexibility, which took the initial SF allocation and the interference-based adaptive SF allocation in the deployment phase [21].

#### 1.1.2. Transmission Power (TP)

The TP is also one of the optimization parameters in LoRa networks. The iterative approach has been usually used to allocate the minimum power until the minimum power index is reached, then the iterative allocation of the maximum power until the maximum power index is reached, iterating between the two to allocate the remaining TP [22]. By monitoring the channel status and link quality of the network, the dynamic optimization of transmission power was achieved based on feedback mechanisms, maximizing the efficiency of data packet transmission [23]. The mathematical model was established for LoRa networks, which considered factors such as energy efficiency, signal quality, and network capacity. The numerical methods were employed to implement an optimal transmission power control algorithm [24]. To achieve a fair data rate independent of the gateway distance, it is necessary to avoid excessive TP to reduce energy consumption.

#### 1.1.3. Adaptive Data Rate

To optimize the adaptive data rate, some works proposed a greedy algorithm, which designed an enhanced greedy adaptive data rate algorithm with coding rate adaptation to optimize the trade-off between delivery ratio and energy consumption [25]. Moreover, some guarantee parameters were dynamically selected to compensate for the error of the adaptive data rate channel condition estimation to meet the reference data extraction rate [26]. In adaptive data rate technology, the gradient projection method was considered for addressing contention issues caused by data rate [27]. Based on the link, the binary search algorithm was used to configure the transmission parameters of terminal nodes, achieving lower complexity [28]. The terminal node status was determined, and a Kalman filter was applied to estimate the signal-to-noise ratio for mobile terminals, thus determining the terminal transmission parameters [29].

#### 1.1.4. Packet Distribution Ratio

Some other works adopted reinforcement learning methods in the packet distribution ratio allocation strategy, and the resource allocation problem was modeled as a Multi-Armed Bandit (MAB). Through a two-phase algorithm named MIX-MAB algorithm, the terminal can determine its transmission parameters in a distributed mode [30]. Based on collected data from an actual network, including link quality and environmental conditions, the packet distribution ratio model was developed to calculate the successful transmission rate of data packets in LoRa networks [31].

### 1.2. Motivation and Contributions

The problem of near-far effect is one of the causes of unfairness in the LoRa network. In wireless communication, the received power varies between different locations due to the influence of path loss, fading, and other effects during data transmission. For the general case, we assume that the receive power at the receiving end close to the transmitter source may be higher than the distant receiving end, but it is not necessarily the maximum. This situation can affect the capture effect of the LoRa network. Therefore, the received power of all terminals needs to be balanced to achieve a fair data rate, regardless of their distance from the gateway [22]. However, the optimization of resource allocation in the LoRa network focused on a single parameter such as the spreading factor, and the optimization goal is limited to a single goal such as optimizing the terminal adaptive data rate. There is still a great lack of tools, methods, and models for multi-radio parameter, multi-objective optimization. In general, traditional dynamic programming and Lagrangian relaxation methods often face the curse of dimensionality when solving problems. With the development and application of commercial solution software, Mixed Integer Linear Programming (MILP) has gradually shown its good advantages [32]. In the MILP model, optimization problems can usually be modeled as seeking solutions that satisfy certain optimal properties under certain conditions [33]. These problems can be expressed by a set of decision variables, a set of constraints, and a set of objective functions. The main advantage is that it can utilize mature computing tools, and the solution methods are diverse, with controllable solution accuracy and calculation time [34].

In this paper, we use Mixed Integer Linear Programming (MILP) to optimize LoRa radio parameter configuration to improve LoRa network performance. Starting from the management of wireless resources by the gateway, we propose an optimal framework for the joint allocation of SF and TP. To maximize the data extraction rate in the LoRa network and ensure the lowest energy consumption, we develop and optimize a solution in terms of mixed integer linear programming formulations to solve problems.

Our contribution is to solve the resource allocation optimization problem in the LoRa network uplink transmission system. By establishing a multi-objective function with the lowest packet collision probability and the lowest network energy consumption, we propose an optimal joint allocation of SF and TP. In this scheme, the radio parameter assignment problem is constructed as a MILP model and solved with the aid of the optimization solver Gurobi [35]. Finally, we perform a simulation analysis of the network performance based on the obtained optimal solution.

The main contributions of this paper are summarized as follows:

- According to the Aloha protocol mechanism adopted in the LoRa network, we classify data packets collision into three theorems, namely, no conflict, conflict avoidance, and conflict occurrence. The above theorems can provide a basis for analyzing the collision effect and capturing the effect of packets in the LoRa network.
- Against the singleness of parameter allocation of the existing LoRa network, we propose the improvement idea of multi-parameter and multi-objective optimization, and transform the problem into a Mixed Integer Linear Programming (MILP) model. The proposal of this model fundamentally eliminates the defect of single parameter optimization in the LoRa network.
- Since the MILP model is a non-convex optimization problem, which is difficult to solve in terms of time and efficiency, we propose the heuristic fusion algorithm, which uses the combination of Gurobi mathematical solver and the heuristic genetic algorithm. We input the better solution obtained by the heuristic genetic algorithm into the solver as the initial solution of Gurobi, and get the final results through Gurobi.
- Numerical results demonstrate that the proposed algorithm can significantly reduce the number of data packet collisions and effectively reduce network energy consumption, with good fairness among terminals. Compared with the dynamic radio parameter allocation strategy, including Genetic Algorithm (GA) and Approximate Average Packet Airtime Algorithm (AAPA) [36], the average increase in data extraction rate is 5.2%, and the energy consumption is reduced by an average of 41.5%. Compared to traditional allocation strategies, including minimum airtime allocation, equal distribution allocation, and random allocation, the data extraction rate increases by an average of 14.3%, and energy consumption decreases by an average of 23.7%.

### 1.3. Organization

The rest of this paper is organized as follows. The LoRa network collision effect is analyzed in Section 2, including the transmission model and collision analysis. The LoRa network resource allocation optimization is introduced in Section 3, including the problem formulation and the MILP solution algorithm. Section 4 provides the simulation results. The conclusions are finally summarized in Section 5.

## 2. LoRa Network Collision Effect

### 2.1. Transmission Model

The transmission rate of the LoRa wireless network is given by [37]:

(1) $$\begin{array}{c} R_{b}=SF\times \frac{\left[\frac{4}{4+CR}\right]}{\left[\frac{2^{SF}}{{\left.BW\right|}_{Hz}}\right]}\;\mathrm{bps} \end{array}$$

where $R_{b}$ is the data rate, spreading factor SF ∈ {7, 8, 9, 10, 11, 12}, coding rate CR ∈ {1, 2, 3, 4}, bandwidth BW ∈ {125, 250, 500}. The LoRa network transmission rate varies due to the radio parameter selection of each terminal. When BW and CR are constant, the increase of SF can make the signal resist greater interference, thereby increasing the transmission range, but at the same time, it will also increase the airtime of the data packet, which will lead to a decrease in data rate and increase energy consumption, so a trade-off should be made between transmission range and energy consumption by changing SF. In particular, data packet transmission time $T_{s}$ consists of the preamble transmission time $T_{preamble}$ and the payload transmission time $T_{payload}$, and satisfies the following equation:

(2) $$\begin{array}{c} T_{s}=T_{preamble}+T_{payload} \end{array}$$

where $T_{preamble}$ and $T_{payload}$, respectively, satisfy the following relationship [15]:

(3) $$\begin{array}{c} T_{preamble}=\left(n_{preamble}+4.25\right)\times T_{sym} \end{array}$$

(4) $$\begin{array}{c} T_{payload}=payloadSymbNb\times T_{sym} \end{array}$$

where $T_{sym}$ represents the time required to send a single symbol, $n_{preamble}$ is the length of the preamble, payloadSymbNb is the number of symbols that make up the packet payload and header, $T_{sym}$ and $n_{preamble}$ respectively satisfy the following formula [37]:

(5) $$\begin{array}{c} T_{\mathrm{sym}}=\frac{2^{SF}}{BW} \end{array}$$

(6) $$\begin{array}{c} payloadSymbNb=8+\max\left(ceil\left(\frac{8PL-4SF+28+16-20H}{4(SF-2DE)}\right)\left(CR+4\right),0\right) \end{array}$$

where PL represents the length of the payload, *H* represents the selected header type, DE represents whether low-rate optimization is used during data transmission, and ceil is the rounding function.

To analyze the dense LoRa network deployment scenario, we adopt the log distance propagation model [37]:

(7) $$\begin{array}{c} L_{pl}\left(d\right)=\bar{L_{pl}}\left(d_{0}\right)+10\gamma log\frac{d}{d_{0}}+X_{\sigma } \end{array}$$

where $L_{pl}\left(d\right)$ is the path loss, $\bar{L_{pl}}\left(d_{0}\right)$ is the mean path loss at the reference distance $d_{0}$, γ is the path loss index, and $X_{\sigma }$ is a normal distribution with zero mean, that is $X_{\sigma }∼N\left(0,\sigma^{2}\right)$.

### 2.2. Collision Analysis

We assume that the terminal in LoRa network adopts the Aloha protocol, which does not require gateway authorization and allocation of time-frequency resources, and can send data packets when needed. The Aloha protocol can achieve low power consumption and low latency of the terminal when the network load is low, but in the case of massive terminal access, the data packets sent by the terminal will generate a large number of conflicts, and the success rate of data transmission will drop sharply.

**Figure 2:**
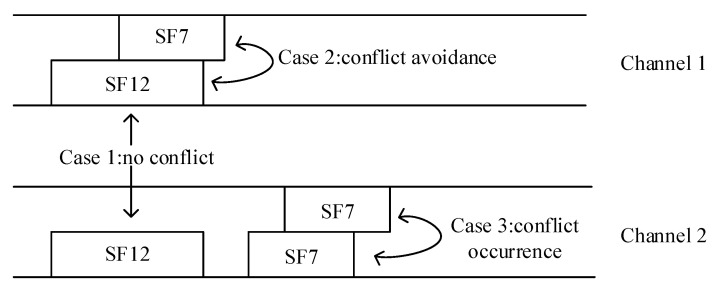
Aloha multiple access control based on LoRaWAN.

At the receiving end, only when the packet received power $P_{rx}$ is not lower than the receiving sensitivity $RX_{sen}$, the data packet can be received successfully, and $RX_{sen}$ depends on the combination of SF and BW [37,38], which satisfies:

(8) $$\begin{array}{c} P_{rx}\geq RX_{sen} \end{array}$$

where $P_{rx}=P_{tx}+G-L-L_{pl}$, and $P_{tx}$ is LoRa terminal transmission power, *G* is antenna gain, *L* is transmitter signal loss, and $L_{pl}$ is the path loss between the transmitter and receiver. However, in real LoRa networks, SF is not perfectly orthogonal [39,40,41]. The impact due to this imperfect orthogonality has been shown to depend on the LoRa transceiver, the signal-to-noise ratio, and the number of deployed terminals [40]. In this paper, we assume that SF is orthogonal. As shown in Figure 2, assuming that the available SFs are SF7 and SF12, consider the following three theorems:

**Theorem 1.**  *(No Conflict) Two data packets using the same SF arrive at the same time, and because they are on different channels, they can be successfully decoded.*

**Proof of Theorem 1.**  Assume that both data packets *a* and *b* use the same spreading factor SF12 and arrive at the same time. Define *a* on channel C1 and *b* on channel C2. Because C1 and C2 belong to different channels and are disjoint with each other, C1 is not equal to C2, so there is no chance of collision between the data packets located in C1 and C2, so the two data packets *a* and *b* do not interfere with each other and can be successfully decoded.    □

**Theorem 2.**  *(Conflict Avoidance) Since the SFs are mutually orthogonal, collisions can be avoided even if multiple terminals use the same channel.*

**Proof of Theorem 2.**  Assume that terminal D1 uses spreading factor SF7, terminal D2 uses spreading factor SF12, and both D1 and D2 occupy the same channel C1. Because the spreading factors SF7 and SF12 are orthogonal to each other, that is, SF7∩SF12=0, the data packets of terminal D1 and terminal D2 do not interfere with each other and can be successfully decoded.    □

**Theorem 3.**  *(Conflict Occurrence) Collisions occur because data packets using the same SF overlap in time on the same channel.*

**Proof of Theorem 3.**  Assume that both packets *a* and *b* use the same spreading factor SF7, *a*, and *b* are located on the same channel C2 and overlap in time. Because the spreading factors SF7 and SF7 are the same, they do not satisfy the mutually orthogonal condition, that is, SF7∩SF7≠0, so the two data packets of *a* and *b* on the same channel will interfere with each other and cause collisions.    □

To sum up, when two or more data packets have the same carrier frequency, use the same SF, and have overlapping arrival times, it can be considered that a collision will occur.

In addition, when a collision occurs, if the difference between the received power of two data packets is not less than 6dB, a capture effect will occur, the data packet with higher power is received, and the other is lost. When the difference is less than 6dB, the receiver will constantly switch between the two packets, unable to efficiently decode either packet [42].

## 3. LoRa Network Resource Allocation Optimization

### 3.1. Problem Formulation

How to effectively optimize the resource allocation of the LoRa network is the key issue to improve the LoRa transmission performance. Therefore, we establish the relevant constraints between different radio parameters to make the trade-off between transmission distance, energy loss, and anti-interference. We consider a LoRa network system with a single gateway and *N* terminals. The gateway is located in the center, and the LoRa terminals are randomly distributed around the gateway. The average data generation rate of each LoRa terminal is λ, the average transmission length is ξ bytes, and the transmission time of the data packet is $T_{s}$.

Based on the access characteristics of the Aloha protocol, we define the average collision probability $P_{coll}$ as follows:

(9) $$\begin{array}{c} P_{coll}=1-\frac{1}{N}\sum_{i=1}^{N}e^{\left(-2G_{s,i}\right)} \end{array}$$

where $G_{s,i}$ represents the traffic load. Moreover, the average number of packets arriving at the channel within the packet transmission time $T_{s,i}$ of the terminal *i* determined by SF is recorded as $\lambda \times N_{i}$, where $N_{i}$ is the number of terminals using the same SF as terminal *i*, then $G_{s,i}$ can be expressed as:

(10) $$\begin{array}{c} G_{s,i}=T_{s,i}\times \lambda \times N_{i} \end{array}$$

It can be seen from Equation (10) that the more data packets arrive at the network, the greater the network load, and the higher the possibility of data packet collision. Terminals of the same SF are generally considered to interfere with each other, but given the capture effect, the following optimizations can be made:

(11) $$\begin{array}{c} C_{pwr}\left(i,j\right)=\left\{\begin{array}{cc} 1, & \left|P_{rx}^{i}-P_{rx}^{j}\right|\leq 6\\ 0, & otherwise \end{array}\right. \end{array}$$

where $C_{pwr}\left(i,j\right)$ indicates whether there will be a conflict between terminals *i* and *j* with the same SF, $P_{\mathrm{rx}}^{i}$ and $P_{\mathrm{rx}}^{j}$ represent the received power of the two terminals respectively. Therefore, the total number of conflicting terminals can be defined as:

(12) $$\begin{array}{c} N_{i}=\sum_{j\in N,j\neq i}C_{pwr(ij)}^{i} \end{array}$$

where $N_{i}$ is always not greater than the total number of terminals using SF.

When the number of periodically transmitted terminals increases dramatically, the collision problem of data packets in the LoRa network will become a serious challenge. While ensuring the low energy consumption of the LoRa network, how to reduce data packet loss is an urgent problem to be solved. Due to the capture effect, the received power level will determine whether the data packet is decoded when the data packet collision occurs. Therefore, we define network energy consumption (NEC) as follows:

(13) $$\begin{array}{c} NEC=\sum_{i=1}^{N}T_{s,i}\times P_{tx,i}. \end{array}$$

We set the optimization goal as the lowest network energy consumption while minimizing the collision rate, as follows:

(14) $$\begin{array}{cc} & \min\;\min P_{coll}\;NEC\\ s.t. & SF\in \{7,8,9,10,11,12\},\\ & TP\in \{2,5,8,11,14\}. \end{array}$$

The key parameters to solve the LoRa network optimization problem are the data packet transmission time and received power. On the one hand, a larger transmission rate can be obtained by using a smaller SF, resulting in a shorter transmission time. On the other hand, a higher receiving sensitivity can be obtained by using a larger SF, but the transmission rate is lower and the transmission time is increased. Therefore, it is necessary to find an optimal solution of mutual constraints among received power, transmission time, and the number of SF terminals used.

### 3.2. MILP Solution Algorithm

To model the resource allocation problem of the LoRa network as MILP problem, we define the tuple $W_{i\in \{1\ldots N\}}=\left(sf_{i,SF},tp_{i,TP}\right)$, where SF and TP represent the list of spreading factor and transmission power available to the terminal, respectively. $W_{i}$ is a boolean array, used to indicate whether terminal *i* uses the given SF and TP. Our goal is to reduce the energy consumption of the LoRa network as much as possible under the condition of minimizing the terminal collision probability, where minimizing the terminal collision probability is equivalent to maximizing the data transmission success rate of each (SF,TP) combination. We define that the decision variable of each terminal has two boolean arrays SF and TP, which are used to represent the transmission parameters SF and TP used by the terminal, respectively. We define the objective function as follows:

(15) $$\begin{array}{c} \begin{array}{c} \sum_{tpi\in TP}\sum_{sfj\in SF}\left(U_{tpi,sfj}-U_{tpl,sfk}\right)\\ \sum_{tpi\in TP}\sum_{sfj\in SF}\left(T_{s,sfj}\times N_{tpi,sfj}\right)\\ tpl:tpl\in TP\cap tpi\neq tpl;\\ sfk:sfk\in SF\cap sfj\neq sfk; \end{array} \end{array}$$

where $U_{sf,tp}$ is the utility function of the (SF,TP) combination, and the objective function reflects the minimization of the difference between the utility of each (SF,TP), so that each (SF,TP) has the least load and achieves good fairness among terminals. The utility function is expressed as:

(16) $$\begin{array}{c} U_{tpi,sfj}=N_{tpi,sfj}\times T_{s}\left(sfj\right)\times \lambda ; \end{array}$$

(17) $$\begin{array}{c} U_{tpl,sfk}=N_{tpl,sfk}\times T_{s}\left(sfk\right)\times \lambda \end{array}$$

where $N_{tpi,sfj}$ is the number of terminals using $\left(SF_{j},TP_{i}\right)$, and $N_{tpl,sfk}$ is the number of terminals using $\left(SF_{k},TP_{l}\right)$. To improve the receiving reliability of the system, we use the receiving sensitivity as a constraint condition to manage the selection of spreading factor, so that the terminal can allocate the best parameters under the premise of ensuring that the data packets reach the gateway:

(18) $$\begin{array}{c} P_{der}^{i}=\left\{\begin{array}{cc} 1, & P_{rx}\geq RX_{sen}\left(SF\right)\\ 0, & P_{rx}<RX_{sen}\left(SF\right). \end{array}\right. \end{array}$$

Therefore, $N_{tp_{i},sf_{j}}$, $N_{tp_{l},sf_{k}}$ can be expressed separately as:

(19) $$\begin{array}{cc} & N_{tpi,sfj}=\sum_{i}^{N}tp_{i,tpi}\times sf_{i,sfj}\times P_{\mathrm{der}}^{i} \end{array}$$

(20) $$\begin{array}{cc} & N_{tpl,sfk}=\sum_{i}^{N}tp_{i,tpl}\times sf_{i,sfk}\times P_{\mathrm{der}}^{i}. \end{array}$$

In addition, considering that each terminal is only assigned one SF and one TP, the following constraints exist:

(21) $$\begin{array}{cc} \sum_{tp\in TP}\left(tp_{i,tp}\right)=1 & \forall i:1..N \end{array}$$

(22) $$\begin{array}{cc} \sum_{sf\in SF}\left(sf_{i,sf}\right)=1 & \forall i:1..N. \end{array}$$

Equations (19) and (20) belong to the product of multiple binary variables and is a nonlinear expression. Therefore, we introduce auxiliary variables for linearization transformation for terminal i:

(23) $$\begin{array}{c} z_{i,tp,sf}=tp_{i,tp}\times sf_{i,sf}. \end{array}$$

There are the following constraints:

(24a) $$z_{i,tp,sf}\leq tp_{i,tp}$$

(24b) $$z_{i,tp,sf}\leq sf_{i,sf}$$

(24c) $$z_{i,tp,sf}\geq tp_{i,tp}+sf_{i,sf}-1.$$

In the same way:

(25) $$\begin{array}{c} r_{i,tp,sf}=z_{i,tp,sf}\times P_{\mathrm{der}}^{i}. \end{array}$$

The following constraints exist:

(26a) $$r_{i,tp,sf}\leq z_{i,tp,sf}$$

(26b) $$r_{i,tp,sf}\leq P_{\mathrm{der}}^{i}$$

(26c) $$r_{i,tp,sf}\geq z_{i,tp,sf}+P_{\mathrm{der}}^{i}-1.$$

In this paper, by establishing a MILP model, the system performance optimization problem is transformed into a mathematical problem to achieve the allocation of spreading factor and transmission power. In the model, the spreading factor and transmission power are used as decision variables, and the network data extraction rate and network energy consumption are used as objective functions, with constraints introduced. We use the hierarchical sequence method to solve this problem, that is, first obtain the optimal solution set corresponding to the minimum conflict rate under the constraint conditions, and then solve the optimal solution corresponding to the lowest energy consumption under the premise of ensuring the optimal solution of the previous goal. For the MILP model, we use the mathematical solver Gurobi, the heuristic genetic algorithm, and the combined algorithm of the two to solve them separately, and input the better solution obtained by the heuristic genetic algorithm into the solver as the initial solution of Gurobi. The algorithm flow is as shown in Algorithm 1.
**Algorithm 1** Heuristic fusion algorithm.**Input:** Available SF and TP lists,**Output:** Optimal spreading factor and transmission power configuration for each terminal.  1:**Step 1:** Describes the utility function $U_{tp,sf}$, transmission energy consumption, and enter the constraints;  2:**Step 2:** Encode the parameters SF and TP and initialize the population;  3:**Step 3:** Calculate the fitness, and determine whether the stopping conditions are met;  4:**Step 3.1:** If the stop condition is satisfied, the solution (SF,TP) is assigned to the decision variable in Gurobi;  5:**Step 3.1.1:** Resolve to determine the optimal solution (SF,TP);  6:**Step 3.2:** Select, cross, mutate, and repeat step 3 until the stopping conditions are met;

## 4. Simulation Results

To show the performance of different parameter selection strategies, we choose the LoRaSim simulation platform for performance verification [43]. With this tool, we deploy terminals as well as a gateway in a two-dimensional area, assuming that a single gateway can decode concurrent signals on all SFs. The parameters set in the simulation are shown in Table 1.

We use three types of indicators to evaluate the performance of the LoRa network, namely data extraction rate (DER), the number of collisions, and network energy consumption, in addition to evaluating the effectiveness of the model.

It can be found from the analysis that our proposed MILP model is a non-convex optimization problem. Therefore, when using the mathematical solver, we first set a fixed solution time of 3600 s to obtain as good a solution accuracy as possible within this time. For the proposed MILP model, we combined the Gurobi mathematical solver and heuristic genetic algorithm to solve the problem. The parameter allocation strategies we selected include minimum airtime allocation, equal distribution allocation, random allocation, Genetic Algorithm (GA), and Approximate Average Packet Airtime Algorithm (AAPA) [36].

**Figure 3:**
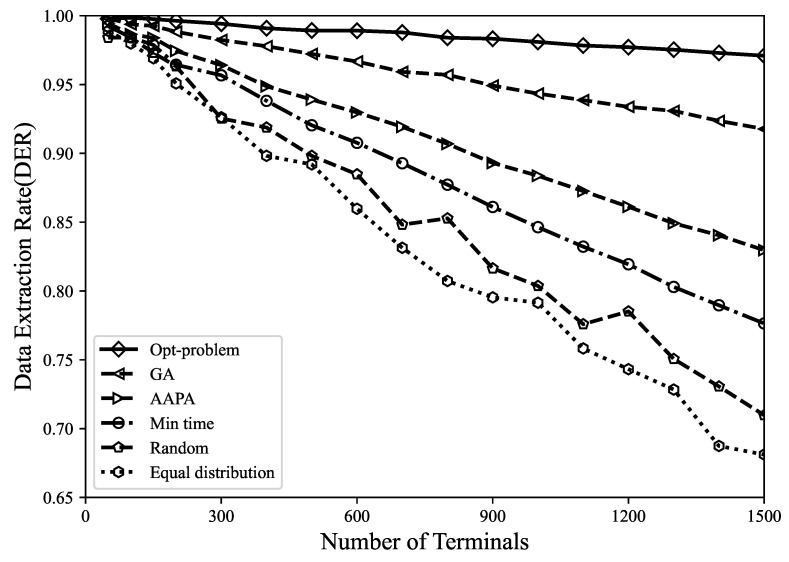
When using different parameter allocation strategies, DER varies with the number of terminals.

*(1) Data extraction rate*: We evaluate the data extraction rate of the LoRa network within the value range between 0∼1. The closer the DER is to 1, the more effective the LoRa deployment will be. The following is the calculation formula of DER:

(27) $$\begin{array}{c} DER=\frac{Nr-C}{Ns} \end{array}$$

where $N_{r}$ represents the number of received packets, *C* represents the number of conflicting packets, and $N_{s}$ represents the number of sent packets. As shown in Figure 3, compared with the genetic algorithm, AAPA algorithm, minimum airtime allocation, random and equal distribution allocation, the DER optimal solution obtained by using the Gurobi tool increased by 2.7%, 7.6%, 10.7%, 14.8%, and 17.3%. It can be found that the genetic algorithm has a poor search ability and low search efficiency in the face of high-dimensional and complex problems, and it is prone to premature convergence problems and falls into local optimal solutions. In addition, random allocation and equal distribution allocation are suitable for the situation with few LoRa network terminals. The disadvantage is that random allocation may have terminals close to the gateway using high SF, thereby increasing the airtime of data packets and increasing the probability of collision, whereas equal distribution allocation only distributes the number of terminals evenly among (SF,TP), without considering the different applicable distances of SF. The model proposed in this paper is a global optimization that considers all terminals in the entire network, providing the optimal spreading factor and precise transmission power values for each terminal. Accurate parameter allocation helps to reduce packet collisions and interference, thereby improving the data extraction rate of the network.

**Figure 4:**
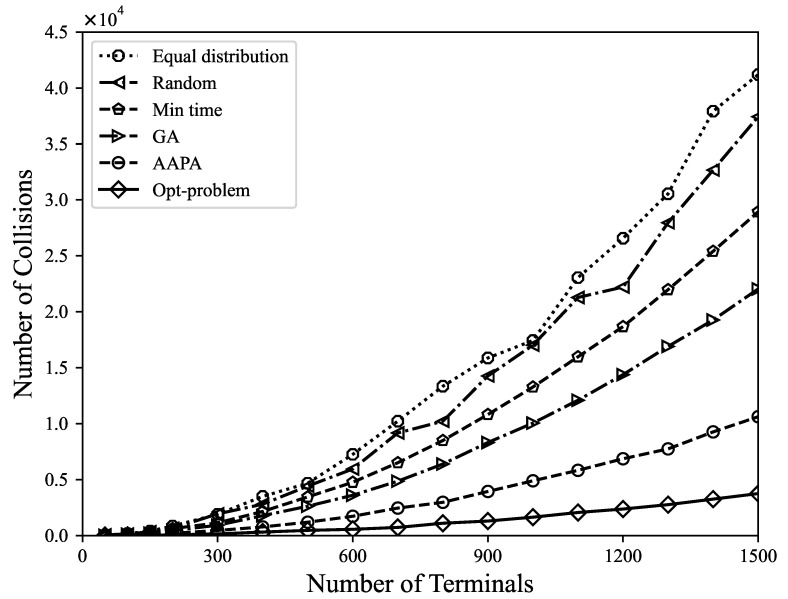
The relationship between the number of packet collisions and the number of terminals.

**Figure 5:**
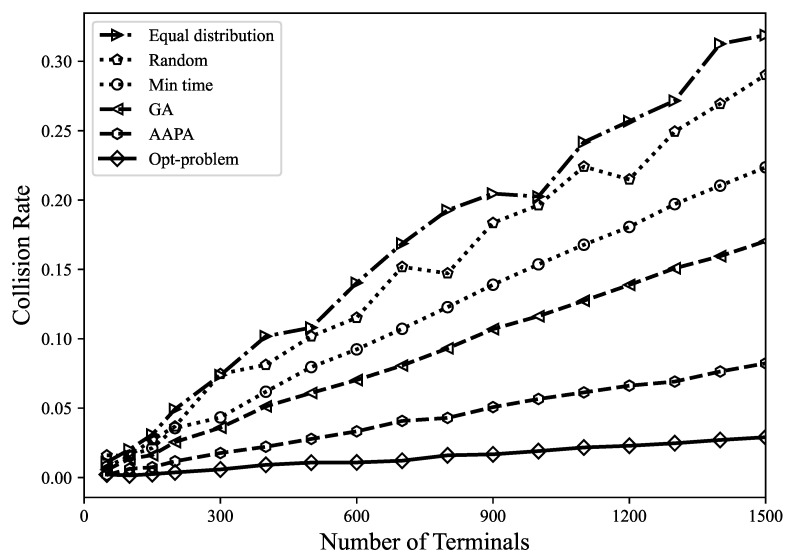
Collision rate among different terminals.

*(2) Number of collisions*: When there are two or more terminals in the LoRa network sending data at the same time, if a collision occurs, the transmitted data may be lost. When two LoRa transmissions occur at the same time, by analyzing the SF, TP, energy, and time conditions, it is determined that the receiving end can simultaneously decode the received packets. From Figure 4 and Figure 5, it can be found that the number of data packet collisions and the collision rate are under different parameter allocation strategies. It shows that the number of data packet collisions caused by the Gurobi optimization strategy is the lowest, which is far lower than other parameter allocation strategies. After optimization, the network strives to ensure that each terminal has an equal transmission opportunity by maximizing the fairness among terminals, thereby balancing the utilization of resources among different terminals. When resource allocation is more balanced, the transmission timings of different terminals are less likely to overlap, thereby reducing packet collisions.

**Figure 6:**
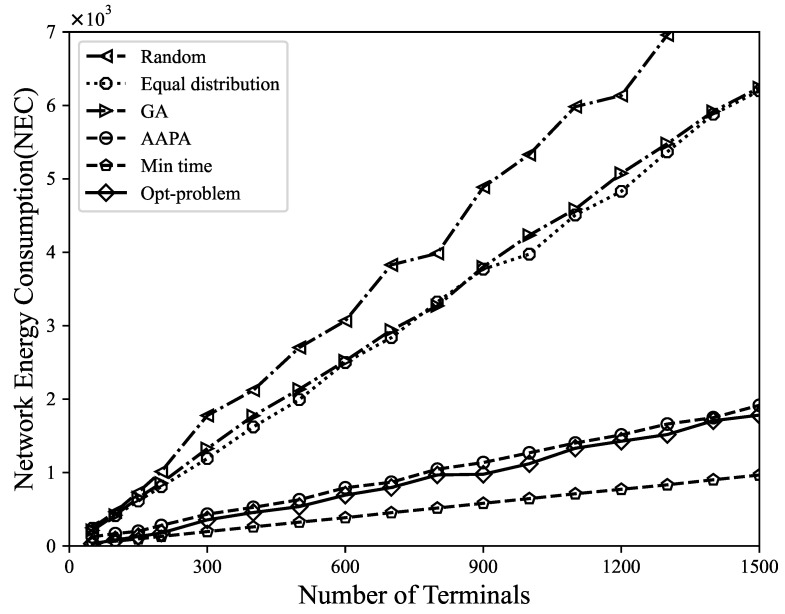
The relationship between network energy consumption and number of terminals.

*(3) Network energy consumption*: The NEC is defined as the energy consumed by the network to successfully extract information. Generally, the energy consumption of the LoRa terminal depends mostly on the energy consumption of the transceiver. Since terminals are mostly battery-powered, the energy consumption of the transmission must be kept to a minimum. Figure 6 shows the trend of NEC changing with the number of terminals. For every increase in SF of 1, the transmission rate is halved, the transmission duration is doubled, and the final energy consumption is doubled [42]. It can be seen from the results that using Gurobi to solve the MILP model can not only maximize the DER of the network, but also effectively ensure the lowest level of energy consumption. The reason is that packet collisions may cause data transmission failures and retransmissions, but this not only consumes network bandwidth and resources, but also consumes additional energy, affecting the overall efficiency of the network. By selecting the appropriate spreading factor and transmission power, packet collisions and packet retransmissions can be reduced, enabling the network to more effectively utilize limited resources and improve terminal energy efficiency. The energy consumption caused by random allocation, equal distribution allocation, genetic algorithm, AAPA algorithm, and minimum airtime allocation is 8.3 times, 6.4 times, 6.5 times, 1.9 times, and 1.8 times the Gurobi optimization strategy, respectively.

**Figure 7:**
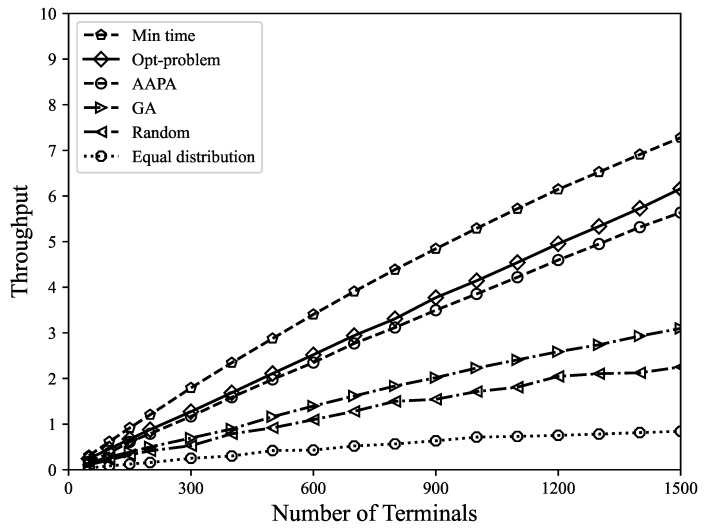
When using different parameter allocation strategies, throughput varies with the number of terminals.

*(4) Throughput*: Due to the low power consumption characteristics of LoRa, high throughput can be achieved through appropriate parameter selection, as selecting a lower SF can improve the transmission rate, whereas selecting the appropriate transmission power can balance packet quality and energy consumption. The model proposed in this paper can improve the data extraction rate while minimizing energy consumption. As shown in Figure 7, the model proposed in this paper also has good performance in throughput. This model enables terminals in the network to achieve the optimal balance between data transmission rate and energy consumption, and can accurately determine the optimal SF and transmission power of each terminal. The parameters of terminals can be dynamically adjusted based on network status and requirements for global optimization, which helps to maximize the performance of the entire network and maintain high throughput. In the minimum airtime allocation, all terminals use the minimum spreading factor, which means that the signal bandwidth is narrow and more data can be transmitted, resulting in high network throughput. Random allocation, equal distribution allocation, and genetic algorithm have certain uncertainties in parameter selection, and cannot achieve global optimization results.

**Figure 8:**
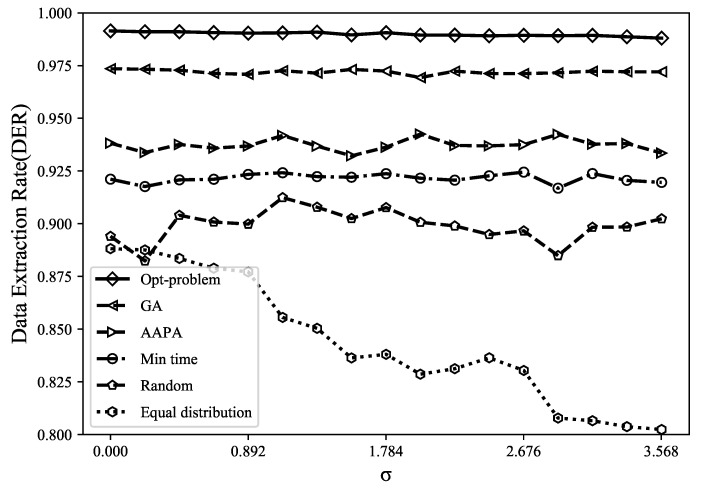
DER under different channel quality.

**Figure 9:**
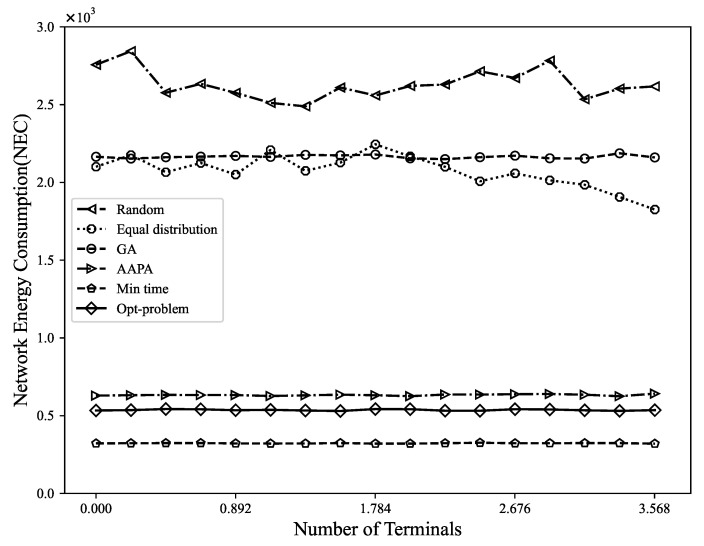
Energy consumption under different channel quality.

*(5) Effectiveness of the MILP model*: We compare the performance of the MILP model under different channel qualities, and the model shows good applicability. From Figure 8 and Figure 9, it can be found that changing σ in the path loss model will hardly affect the DER and energy consumption of the Gurobi optimization parameter allocation strategy, whereas the random allocation and equal distribution allocation strategies are greatly affected by channel interference. This model has dynamic adaptability, allowing terminal parameters to be optimized based on network status and requirements to adapt to worse channel conditions. In addition, based on mathematical programming methods, this model can obtain reliable parameter decisions and maintain stable performance.

**Figure 10:**
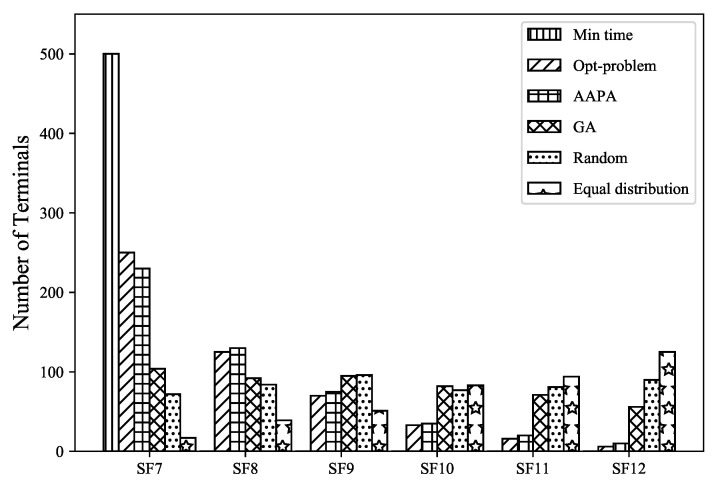
Distribution of each spreading factor.

Furthermore, we take 500 terminals as an example, Figure 10 shows the selection result of SF in the case of a single gateway and multiple terminals. The Gurobi allocation strategy is to select the SF that satisfies the constraints as small as possible, which means that it can achieve lower data packet transmission time, reduce the possibility of collision, and at the same time, it can also achieve network energy consumption under the premise of without reducing the data extraction rate. According to the data packet transmission time formula, the transmission time is the smallest when the BW and CR are fixed and SF equals seven. Therefore, when the terminal transmission time is all concentrated in SF7 in the minimum airtime allocation scheme, the probability of data packet collision is also greatly increased.

**Figure 11:**
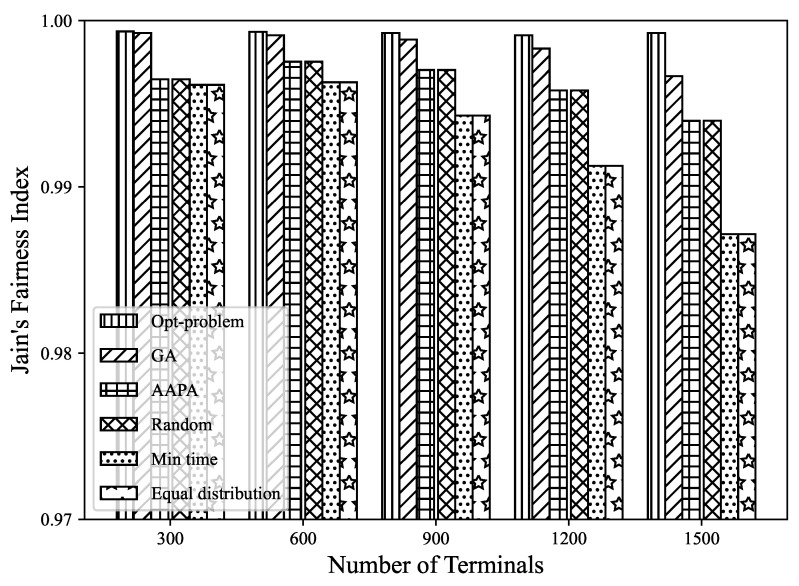
The relationship between the fairness index and the number of terminals.

To evaluate the fairness level of different algorithms, the Jain’s fairness index is introduced as follows [44]:

(28) $$\begin{array}{c} \zeta =\frac{{\left(\sum_{i=1}^{N}DER_{i}\right)}^{2}}{N\sum_{i=1}^{N}DER_{i}^{2}} \end{array}$$

where *N* is the number of terminals in the LoRa network, and $DER_{i}$ is the data extraction rate of terminal *i*. The range of the fairness index is 0∼1, and the higher the value, the fairer the terminals are. Under the assumption that SF is completely orthogonal and the capture effect is considered, the results for different numbers of terminals are shown in Figure 11. It can be seen that regardless of the number of terminals, the fairness index of the proposed model is close to 1, showing good fairness among most terminals in the LoRa network. This model has the characteristic of global optimization, which means that it can simultaneously consider the mutual influence between multiple terminals, which helps to avoid excessive concentration of resources on certain terminals. In Equation (15), the optimization objective is transformed into minimizing the difference in the utility function of nodes, thereby improving overall fairness. However, in the comparison scheme, as the number of terminals increases and the conflicts increase sharply, the fairness index will decrease obviously, which reflects the effectiveness of the MILP model.

**Figure 12:**
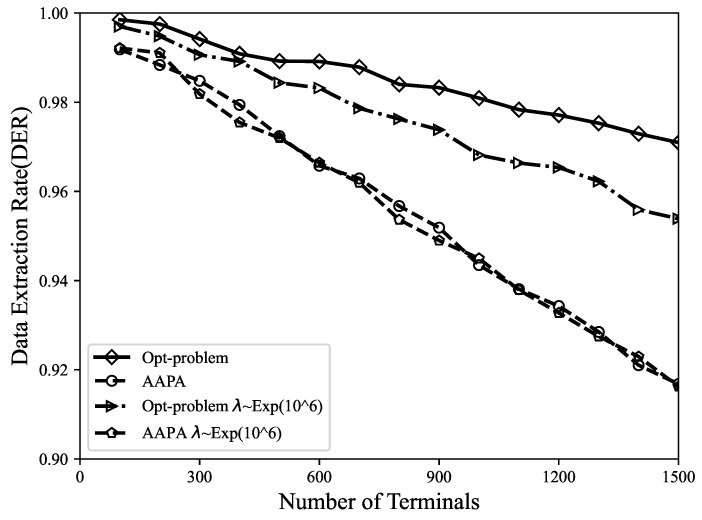
Comparison of data extraction rate with different λ settings.

In Equations (16) and (17), the data packet generation probability λ is fixed. Further, in order to analyze the effect of changes in λ on the algorithms, we assume that the time interval between LoRa terminal data packet transmissions follows a Poisson distribution, and the results shown in Figure 12 are obtained. It can be found that when the packet generation intervals in the network obey the Poisson distribution, the data packet extraction rate decreases synchronously. In this scenario, the proposed algorithm still outperforms the AAPA algorithm. The randomness of the Poisson distribution provides a more realistic simulation of packet generation, but may also increase the probability of data packet collisions and collisions, which demonstrates the excellent performance of the proposed MILP model in LoRa networks.

**Figure 13:**
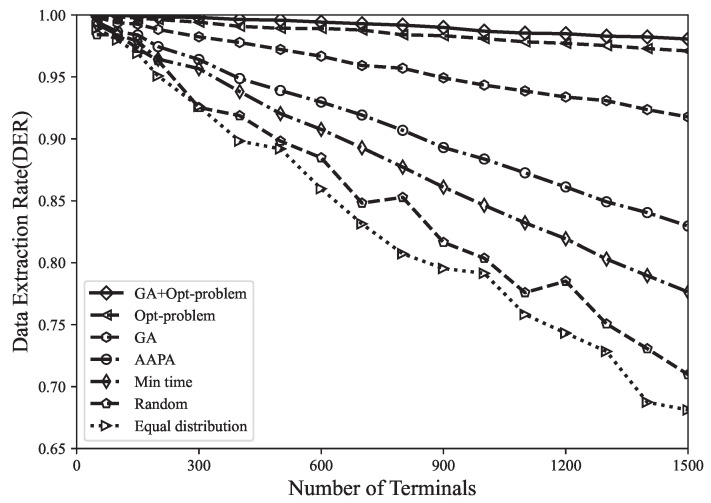
Heuristic fusion algorithm compared with other allocation strategies.

Moreover, when using the Gurobi tool to solve the MILP problem, to further improve the solution accuracy and take into account the solution efficiency factor, the optimal solution of the model obtained by the heuristic algorithm is used as the initial calculation solution of Gurobi. Figure 13 is a comparison of DER under the fusion algorithm, Gurobi without an initial solution, and the heuristic algorithm. It can be found that using the hybrid solution combining the heuristic algorithm and Gurobi, the quality of the obtained solution is higher, and the data extraction rate is further improved. The computational complexity of the model is $O(2^{K}\times B^{M})$, where *K* is the number of decision variables, *B* is the number of binary decision variables, and *M* is the number of constraints. In large-scale terminal and massive machine communication scenarios, it usually requires a lot of computing power for the resource allocation in LoRa networks to be modeled as MILP. This may lead to delayed responses, adversely affecting time-sensitive service operations. In addition, as the network size increases, the scalability of the model may be limited.

## 5. Conclusions

Aiming at the resource allocation optimization problem in the LoRa network uplink transmission system, we proposed an optimal scheme for the joint allocation of spreading factor and transmission power, constructed the radio parameter allocation problem as a MILP model, and solved it with the help of the optimization solver Gurobi. Compared with other algorithms, the parameter allocation we proposed has a higher data extraction rate, and can significantly reduce the number of data packet collisions during transmission, while effectively reducing network energy consumption and increasing network throughput. In the face of changing channel quality, the model can maintain a certain level of stability, and still exhibits good performance under different data generation rates. In particular, the model was evaluated by a fairness index, which verified good fairness among terminals. Finally, the optimal solution obtained by the heuristic algorithm was used as the initial solution of the MILP problem for re-simulation to obtain higher-quality resource allocation and further improved the data extraction rate. Through the scheme proposed in this paper, the performance of the LoRa network can be effectively improved, and the conflict problem in the transmission process can be reduced.

## Figures and Tables

**Table 1:** Simulation parameters.

Parameter	Value	Unit
Spreading Factor SF	7∼12	None
Transmission Power TP	2,5,8,11,14	dBm
Carrier Frequency CF	486	MHz
Bandwidth BW	125	kHz
Coding Rate CR	1	None
Reference Distance $d_{0}$	40	m
Path Loss Index γ	2.08	None
Average Path Loss $\bar{L_{\mathrm{pl}}}\left(d_{0}\right)$	127.41	dB
Payload Length ξ	20	bytes
Number of Gateways	1	None
The Furthest Deployment Distance	1	km
Packet Transmission Period	$10^{6}$	s

## Data Availability

Not applicable.
